# Targeting the membrane-anchored serine protease testisin with a novel engineered anthrax toxin prodrug to kill tumor cells and reduce tumor burden

**DOI:** 10.18632/oncotarget.5214

**Published:** 2015-09-15

**Authors:** Erik W. Martin, Marguerite S. Buzza, Kathryn H. Driesbaugh, Shihui Liu, Yolanda M. Fortenberry, Stephen H. Leppla, Toni M. Antalis

**Affiliations:** ^1^ Center for Vascular and Inflammatory Diseases and the Department of Physiology, University of Maryland School of Medicine, Baltimore, MD 21201, USA; ^2^ National Institute of Allergy and Infectious Diseases, National Institutes of Health, Bethesda, MD 20892, USA; ^3^ Division of Pediatric Hematology, Johns Hopkins University School of Medicine, Baltimore, MD 21205, USA

**Keywords:** anthrax toxin, membrane-anchored serine protease, testisin, hepsin, prodrug

## Abstract

The membrane-anchored serine proteases are a unique group of trypsin-like serine proteases that are tethered to the cell surface via transmembrane domains or glycosyl-phosphatidylinositol-anchors. Overexpressed in tumors, with pro-tumorigenic properties, they are attractive targets for protease-activated prodrug-like anti-tumor therapies. Here, we sought to engineer anthrax toxin protective antigen (PrAg), which is proteolytically activated on the cell surface by the proprotein convertase furin to instead be activated by tumor cell-expressed membrane-anchored serine proteases to function as a tumoricidal agent. PrAg's native activation sequence was mutated to a sequence derived from protein C inhibitor (PCI) that can be cleaved by membrane-anchored serine proteases, to generate the mutant protein PrAg-PCIS. PrAg-PCIS was resistant to furin cleavage *in vitro*, yet cytotoxic to multiple human tumor cell lines when combined with FP59, a chimeric anthrax toxin lethal factor-*Pseudomonas exotoxin* fusion protein. Molecular analyses showed that PrAg-PCIS can be cleaved *in vitro* by several serine proteases including the membrane-anchored serine protease testisin, and mediates increased killing of testisin-expressing tumor cells. Treatment with PrAg-PCIS also potently attenuated the growth of testisin-expressing xenograft tumors in mice. The data indicates PrAg can be engineered to target tumor cell-expressed membrane-anchored serine proteases to function as a potent tumoricidal agent.

## INTRODUCTION

Proteolytic enzymes and their regulatory networks, including cofactors, activators, and endogenous inhibitors, are frequently dysregulated in tumors resulting in increased protease activities that contribute to progression of disease [1]. Manipulation of tumor-promoting proteases is a promising approach for the development of anti-tumor therapies [2, 3]. While the targeting of proteases has been approached in several ways [4], prodrug-like protease substrates that target active over-expressed proteases are an extremely efficient approach to increase selectivity and efficacy while reducing off-target effects [5].

Anthrax toxins requiring proteolytic activation have been engineered to target tumor-overexpressed proteases. Anthrax toxin is a cytotoxic pore-forming exotoxin secreted by *Bacillus anthracis*. Consisting of protective antigen (PrAg), lethal factor (LF), and edema factor (EF), the toxin (the combination of PrAg and LF and/or EF) causes cellular cytotoxicity through a well-characterized mechanism [6], whereas individually these proteins are non-toxic. PrAg binds to either of two cell-surface receptors, tumor endothelial marker-8 (TEM8, *ANTXR1*) and capillary morphogenesis gene-2 (CMG2, *ANTXR2*), of which CMG2 is expressed on nearly all cell types. PrAg (83 kDa) bound to its cell-surface receptor(s) is proteolytically cleaved and activated by furin (*FURIN*) or furin-like proprotein convertases in an exposed flexible loop to generate an active C-terminal 63-kDa PrAg fragment. The newly-generated 63-kDa fragment remains receptor bound and catalyzes the formation of a PrAg/receptor oligomer that presents docking sites to enable up to 4 molecules of LF or EF to bind and translocate into the cytosol, through an endosomal PrAg-formed pore, where they have potent cytotoxic effects [7].

As a highly efficient protease-activated protein delivery system, PrAg can be engineered to deliver different payloads into the cytosol in addition to LF and EF [8–14]. Additionally, PrAg can be engineered to be activated specifically by proteases other than furin. Since furin is ubiquitously expressed, it is advantageous to narrow the cellular protease targets for drug delivery applications. Alteration of the furin protease cleavage site within PrAg to amino acid sequences recognized by either urokinase-type plasminogen activator (uPA, *PLAU*) [15], matrix metalloproteinase 2 (*MMP2*), or matrix metalloproteinase 9 (*MMP9*) [16] renders PrAg a potent uPA- or MMP2/9-activated cytotoxin that has been shown to target tumors that overexpress any of these proteases [17–26]. An engineered anthrax inter-complementing toxin has also been created that requires combined activation by these protease systems for function and killing of tumor cells [20, 27].

In addition to their roles in tumor biology, the uPA and MMP protease systems play leading roles in immune regulation and physiological tissue remodeling [4, 28]. Therefore, while these engineered anthrax toxins are effective when used to target tumors *in vivo*, it is possible that paracrine association of the tumor-secreted proteases with other non-tumor cells in or near the tumor microenvironment could contribute to off-target effects of these toxins. The present study was initiated to determine whether targeting of tumor-expressed membrane-tethered serine proteases could enable a highly-specific, more efficient approach for directed tumor cell killing by engineered anthrax toxins. A family of membrane-anchored serine proteases [29, 30] possess domains that tether them directly to the surface of the plasma membrane with their catalytic serine protease domains exposed on the cell surface. Comprised of 21 known members in humans, this family has emerged as being not only structurally unique but also functionally different from the relatively well-characterized secreted serine proteases. Several members of this protease family have restricted cell- and tissue-type specific expression patterns, and loss-of-function studies in mice have revealed that many possess either limited or redundant roles in normal physiology [31–33]. Of significance, many of these enzymes are consistently overexpressed in a wide variety of tumors and have been found to contribute to the tumorigenic properties of tumor cells in cell culture and *in vivo* [34–51]. The cell-surface localization, limited expression patterns, and limited physiological roles of some members of this group of proteases suggest that they may be promising cell-surface targets for anti-tumor therapies.

The membrane-anchored serine protease testisin (*PRSS21*) is synthesized with a 17-amino acid carboxy-terminal hydrophobic extension that is post-transcriptionally modified with a glycosyl-phosphatidylinositol (GPI) linkage that serves to anchor the protease to the extracellular side of the plasma membrane [52–55]. Testisin has remarkably specific tissue distribution, being constitutively expressed in abundance only in spermatocytes, where it has a specific role in male fertility [56–58]. Yet, testisin possesses the characteristics of a Cancer/Testis Antigen (CTA), a group of proteins whose expression is normally restricted to testis, but which are frequently aberrantly activated in tumors [59, 60]. Testisin is strongly overexpressed in human invasive epithelial ovarian cancers, as well as cervical cancers, while being undetectable in normal ovarian or cervical tissues. In an RT-PCR study of ovarian tumors, Shigemasa *et al*. [61] reported that testisin was present in 80–90% of stage 2 or 3 disease. Bignotti *et al*. [62] also found testisin expressed in primary and metastatic ovarian tumors. Overexpression of testisin in ovarian tumor cells results in increased colony formation in soft agar and increased xenograft tumor growth in severe combined immunodeficient (SCID) mice [63]. Its increased expression has also been found to enhance matrigel invasion of cervical cancer cells [64]. Conversely, reduction of endogenous testisin expression via siRNA-mediated knockdown in ovarian and cervical tumor cell lines leads to reduced colony formation, reduced invasion in cell culture, and reduced cellular resistance to the chemotherapy drug adriamycin [63, 64]. The selective expression of testisin by human tumors relative to its normally restricted expression in testis, combined with the relationship of testisin expression to tumorigenic processes, suggests that testisin is an attractive target for anti-tumor therapeutic approaches.

Here, we sought to determine whether the anthrax toxin could be engineered to be activated by testisin overexpressed by tumor cells. We show that replacing eight amino acids flanking the native furin cleavage site within PrAg with a sequence that can be cleaved by testisin abrogates furin activation and generates a potent anti-tumor prodrug toxin. The resultant engineered PrAg-PCIS protein is a testisin substrate that is cleaved and activated by testisin *in vitro* and in cell culture, and has potent anti-tumor cell activity when combined with a recombinant LF-*Pseudomonas* exotoxin based payload (FP59). Moreover, *in vivo* administration of the toxin inhibited growth of established xenograft tumors in mice by inducing tumor necrosis and reducing tumor cell proliferation.

## RESULTS

### Engineering the mutant PrAg-PCIS protein

**Figure 1:**
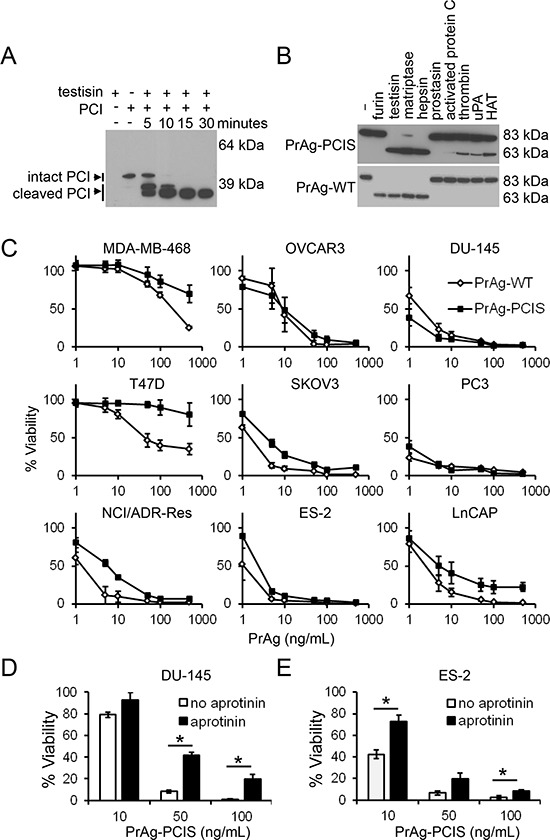
The engineered PrAg-PCIS targets tumor cell serine proteases **A.** PCI is a testisin substrate. Recombinant testisin was incubated with recombinant PCI for various times up to 30 minutes. Individual reactions were stopped at indicated times and immunoblotted using anti-PCI antibody. The blot is representative of two independent experiments. **B.** PrAg-PCIS is resistant to furin cleavage, while PrAg-PCIS and PrAg-WT are susceptible to proteolytic cleavage by various recombinant serine proteases. PrAg-PCIS and PrAg-WT were incubated with furin, the recombinant catalytic domains of membrane-anchored serine proteases, or recombinant pericellular serine proteases for 2.5 hours. Reactions were immunoblotted using anti-PrAg antibody to detect PrAg activation cleavage. The blot is representative of two independent experiments. **C.** PrAg-PCIS and PrAg-WT toxin-induced human tumor cell cytotoxicity. The indicated tumor cell lines were incubated with PrAg proteins (0–500 ng/mL) and FP59 (50 ng/mL) for 48 hours, after which cell viability was evaluated by MTT assay. Values are the means calculated from two independent experiments performed in triplicate. **D.** and **E.** PrAg-PCIS toxin targets serine proteases on the surface of ES-2 and DU-145 tumor cells. Cells were pre-incubated in the presence of a final concentration of 100 μM aprotinin for 30 minutes prior to treatment with the indicated concentrations of PrAg-PCIS and FP59 (50 ng/mL) for 2 hours. Cell viability was evaluated by MTT assay 48 hours later. Values are the means calculated from two independent experiments performed in triplicate. *p < 0.05.

The eight amino acid sequence, ^164^RKKRSTSA, containing the furin cleavage site (furin cleaves the peptide bond between R-S) in the mature wild-type PrAg protein (PrAg-WT) was replaced with the sequence ^164^FTFRSARL (to create PrAg-PCIS) using an overlap PCR strategy. This substrate sequence was derived from a region of protein C inhibitor (PCI, *SERPINA5*), within the reactive center loop and close to the C-terminus, and is known to be cleaved by testisin [65], as we confirmed (Figure 1A), as well as by other serine proteases [66–68]. The mutant and wild-type PrAg cDNAs were expressed in the non-virulent *B. anthracis* strain BH460, and the secreted PrAg proteins purified in high yield using established protocols [69]. Incubation of the PrAg proteins with soluble furin revealed that mutation of the furin cleavage site to that in PrAg-PCIS abrogated furin cleavage, evidenced by its failure to convert the 83-kDa PrAg-PCIS to the activated 63-kDa form (Figure 1B). PrAg-WT was cleaved by furin, as expected (Figure 1B).

### PrAg-PCIS toxin is cytotoxic to a broad range of human tumor cells

The combination of PrAg and FP59, a fusion protein consisting of the PrAg binding domain of LF and the catalytic domain of *Pseudomonas aeruginosa* exotoxin A, has been shown to efficiently kill tumor cells following PrAg activation [70]. When translocated into the cytosol by activated PrAg, FP59 induces cytotoxicity by ADP-ribosylation and inhibition of translation elongation factor-2, resulting in inhibition of protein synthesis and the induction of cell death [70–72]. FP59 does not induce cytotoxicity alone, but must be delivered into cells via an activated PrAg protein to induce cell death. To compare the abilities of PrAg-PCIS and PrAg-WT to be activated by tumor cells and to deliver FP59, cytotoxicity assays were performed on a range of human tumor cell lines after treatment with FP59 in combination with PrAg-PCIS (PrAg-PCIS toxin) or PrAg-WT (PrAg-WT toxin). All tumor cell lines showed a dose-dependent sensitivity to the PrAg-PCIS toxin. In 7 of the 9 tumor lines (NCI/ADR-Res, SKOV3, ES-2, OVCAR3, LnCAP, DU-145, and PC3), the PrAg-PCIS toxin showed potent killing effects at doses similar to the PrAg-WT toxin (Figure 1C). All the cell lines were susceptible to the furin-dependent PrAg-WT, as expected. To determine whether active tumor cell-surface serine proteases were targets of the PrAg-PCIS toxin, ES-2 (ovarian), and DU-145 (prostate) tumor cell lines were pretreated with the cell membrane impermeable serine protease inhibitor aprotinin (Figures 1D, 1E). Serine protease inhibition by aprotinin resulted in significantly reduced PrAg-PCIS toxin-induced cytotoxicity in both cell lines, implicating active cell-surface serine proteases in the mechanism of PrAg-PCIS activation. The incomplete protection from PrAg-PCIS activation conferred by aprotinin could have resulted from partial inhibition of protease activity by aprotinin or toxin activation mediated by serine proteases that are not inhibited by aprotinin.

### Protease selectivity of PrAg-PCIS

**Figure 2:**
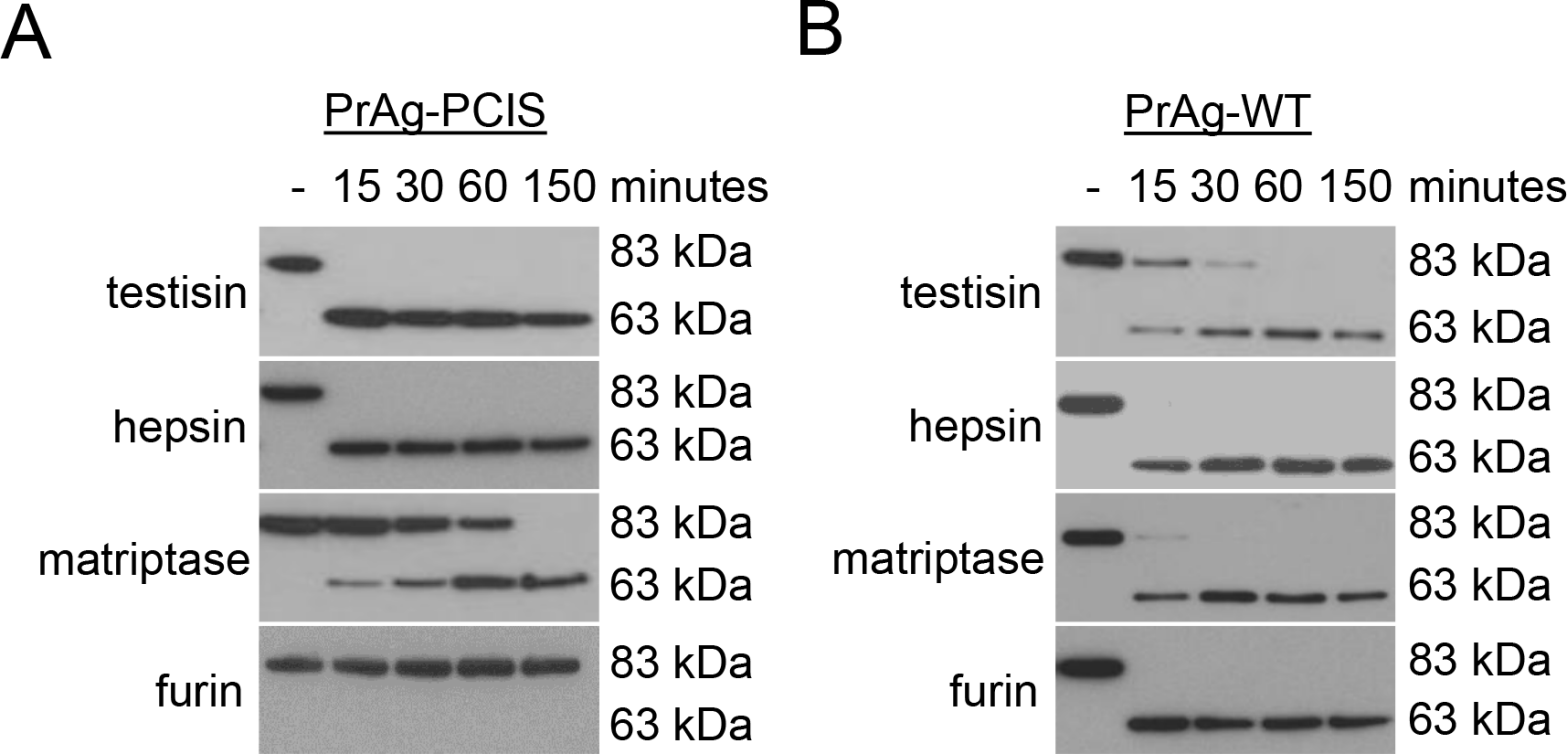
PrAg-PCIS is susceptible to *in vitro* cleavage activation by testisin, hepsin, and matriptase **A.** PrAg-PCIS and **B.** PrAg-WT were incubated with recombinant testisin, hepsin, matriptase, or furin for various intervals up to 2.5 hours. Reactions were immunoblotted using anti-PrAg antibody. Each blot is representative of at least two independent experiments.

Many pericellular proteases, including the membrane-anchored serine proteases, have preferred recognition sequences for substrate cleavage. Yet, there exists promiscuity in sequence recognition and cleavage, particularly with regard to the amino acids adjacent to the cleavage site. Incubation of PrAg-PCIS with the recombinant catalytic domains of several membrane-anchored serine proteases and other potentially reactive pericellular serine proteases resulted in activation cleavage of PrAg-PCIS from the 83-kDa to the 63-kDa form by the membrane-anchored serine proteases testisin, hepsin (*HPN*), matriptase (*ST14*), and to a lesser extent human airway trypsin-like protease (HAT, *TMPRSS11D*) (Figure 1B). As noted previously, PrAg-PCIS was not susceptible to cleavage by soluble furin and showed relatively low susceptibility to cleavage by the secreted serine proteases thrombin (*F2*), activated protein C (aPC, *PROC*), or uPA (Figure 1B). To further investigate the susceptibility of PrAg-PCIS to proteolytic cleavage by testisin, hepsin, and matriptase compared to furin, PrAg-PCIS and PrAg-WT proteins were incubated with the respective recombinant serine protease domains and cleavage was assessed at intervals over time. Testisin and hepsin showed complete activation cleavage of PrAg-PCIS within 15 minutes under the assay conditions, whereas matriptase appeared less effective at PrAg-PCIS cleavage (Figure 2A). As expected, PrAg-WT was effectively cleaved by furin (Figure 2B). Interestingly, PrAg-WT was susceptible to activation cleavage by each of the three serine proteases, testisin, hepsin, and matriptase (Figures 1B, 2B), suggesting a possible role for these membrane-anchored serine proteases in facilitating native PrAg-WT activation and subsequent anthrax toxicity in nature. Analysis of testisin, hepsin, and matriptase mRNA expression in the tumor cell lines susceptible to PrAg-PCIS toxin (Figure 1C) revealed that the tumor cell lines expressed variable levels of some or all of the three proteases, providing the means for PrAg-PCIS activation (Supplementary Figure S1).

**Figure 3:**
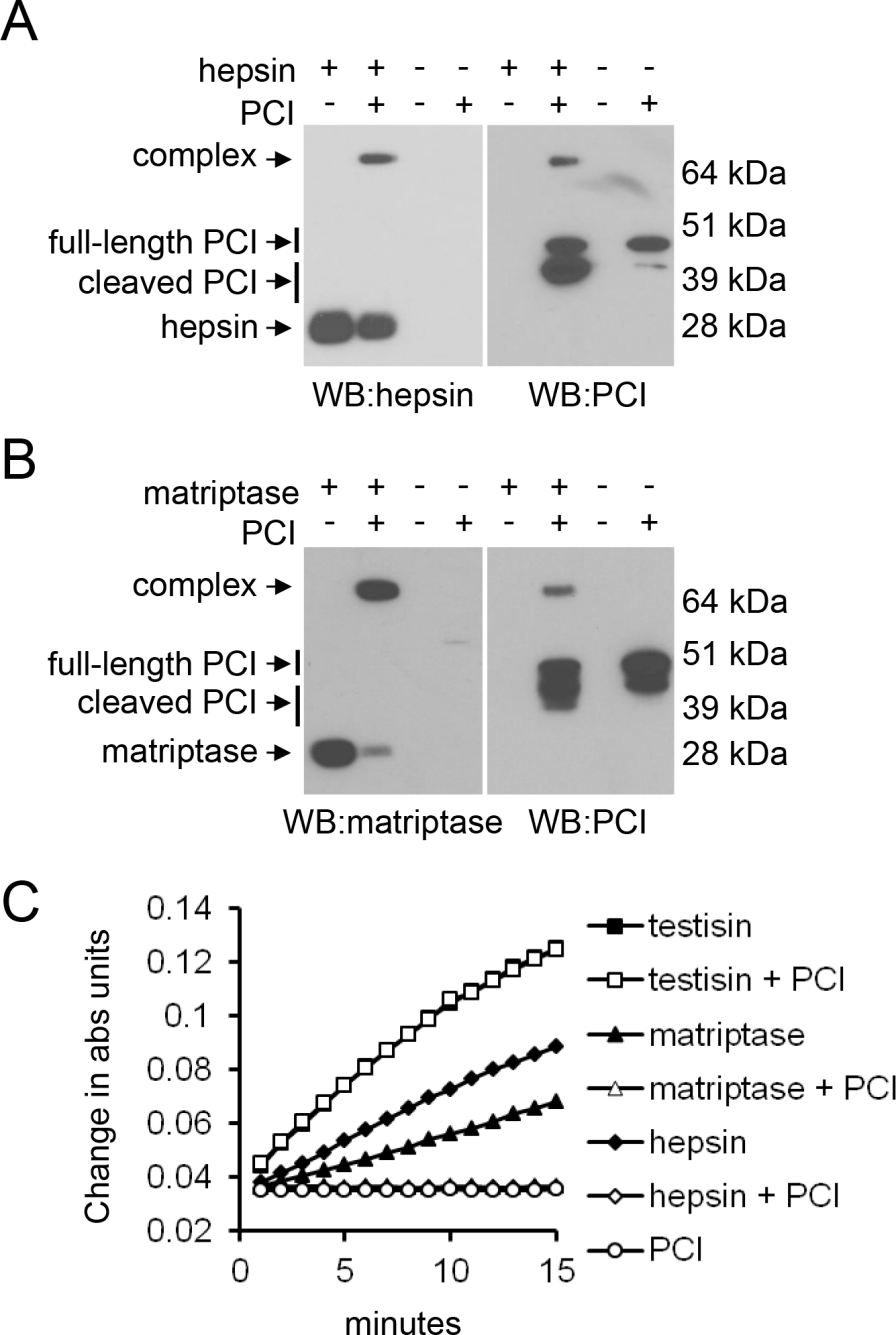
The susceptibility of PrAg-PCIS to proteolytic cleavage by hepsin and matriptase is consistent with their abilities to cleave the RCL of PCI to form protease-serpin inhibitory complexes **A.** Recombinant hepsin or **B.** recombinant matriptase were incubated with PCI, at room temperature prior to immunoblotting with anti-PCI, anti-hepsin, or anti-matriptase antibodies. Full-length PCI, cleaved PCI, and serpin-protease inhibitory complexes are as indicated. Each blot is representative of at least two independent experiments. **C.** PCI inhibits hepsin and matriptase catalytic activities. Recombinant testisin, hepsin, and matriptase were incubated with the peptide substrate, Suc-AAPR-pNA, in the presence or absence of PCI and the changes in absorbance monitored over the course of 15 minutes. The data is representative of at least two independent experiments.

The observation that PrAg-PCIS was susceptible to cleavage by hepsin and matriptase suggested that in addition to native PCI being a substrate of testisin, PCI might be a substrate of these proteases. PCI is a member of the serpin family, whose structure and inhibitory mechanism has been well-characterized [73, 74]. Cleavage of the serpin reactive center loop (RCL) can result in the formation of a protease-inhibitory complex, consisting of PCI covalently bound to the serine protease or production of lower molecular weight cleaved forms of PCI [73, 74]. Incubation of hepsin and matriptase recombinant catalytic domains with PCI resulted in the appearance of cleaved forms of PCI, as well as higher molecular weight complexes representing SDS-resistant serpin-serine protease inhibitory complexes (Figures 3A, 3B). While PCI is a substrate for testisin, inhibitory complexes are not observed when PCI is incubated with testisin (Figure 1A), and, in addition, testisin cleaves PCI at a second site (Figure 1A) as reported previously [65]. Assay of testisin, hepsin, and matriptase peptidase activities using a chromogenic peptide in the absence or presence of PCI confirmed that PCI functions as an inhibitor of hepsin and matriptase catalytic activities, but not testisin (Figure 3C). The abilities of hepsin and matriptase to cleave the RCL of PCI to form protease-serpin complexes, and of PCI to inhibit the catalytic activities of hepsin and matriptase, is consistent with the susceptibility of PrAg-PCIS to proteolytic cleavage by hepsin and matriptase.

### Processing of PrAg-PCIS by cell-expressed GPI-anchored testisin

**Figure 4:**
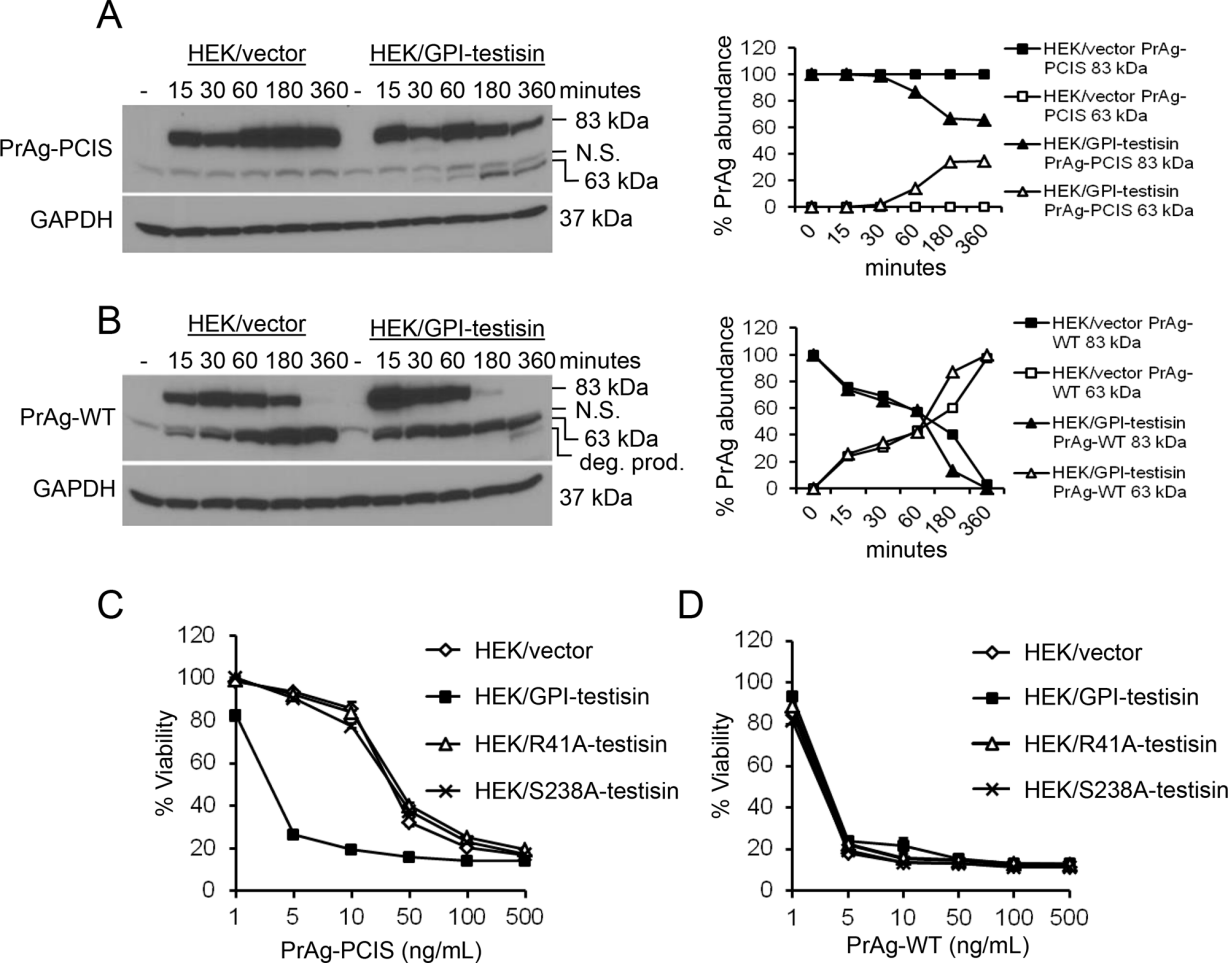
Expression of GPI-anchored testisin in HEK293T cells increases PrAg-PCIS processing and PrAg-PCIS toxin-induced tumor cell killing **A.** Cell-expressed testisin increases processing of PrAg-PCIS. HEK293T cells stably expressing wild-type testisin (HEK/GPI-testisin) or vector alone (HEK/vector) were incubated for up to 6 hours with 500 ng/mL PrAg-PCIS in growth media. At each time point, cells were washed in PBS to remove non-bound proteins and immunoblotted using anti-PrAg antibodies to investigate PrAg cleavage. The blot was reprobed with anti-GAPDH antibody to assess protein loading and is representative of two independent experiments. Densitometric analysis shows cleavage activation of PrAg-PCIS, as indicated by the appearance of the PrAg-PCIS 63-kDa and loss of PrAg-PCIS 83-kDa, in HEK/GPI-testisin cells. **B.** Cell-expressed testisin increases processing of PrAg-WT. HEK/GPI-testisin or HEK/vector cells were treated as in A) and analyzed for PrAg cleavage. The blot was reprobed with anti-GAPDH antibody to assess protein loading and is representative of two independent experiments. Densitometric analysis shows efficient processing of PrAg-WT to the 63-kDa form in both cell lines. In HEK/GPI-testisin cells, an additional band was detected, likely an *in vitro* degradation product. **C.** Active testisin increases PrAg-PCIS toxin-induced cytotoxicity. The indicated cell lines were incubated for 6 hours in growth media with PrAg-PCIS (0–500 ng/mL) and FP59 (50 ng/mL), and then media was replaced with fresh media. Cell viability was assayed 48 hours later by MTT assay. **D.** PrAg-WT toxin-induced cytotoxicity is not dependent on active testisin. The indicated cell lines were treated with PrAg-WT and FP59 and viability measured as in C). MTT assays represent the mean of a total of 6 experiments (3 separate experiments, with triplicate samples, for each of two independent pools of stably-transfected cells).

Following activation cleavage on the cell surface, the cleaved PrAg forms an oligomer which is internalized by the cell. To confirm that testisin anchored on a tumor cell surface can process PrAg-PCIS to an activated form, HEK293T cells stably expressing full-length human testisin (HEK/GPI-testisin) or vector alone (HEK/vector) (Supplementary Figure S2) were exposed to PrAg-PCIS or PrAg-WT for various times up to 6 hours and assayed for the appearance of the 63-kDa activation product. The processing of PrAg-PCIS to the 63-kDa form was detectable in HEK/GPI-testisin cells within 30 minutes and these levels increased with time (Figure 4A). Importantly, PrAg-PCIS was not processed in the absence of testisin in HEK/vector cells (Figure 4A), consistent with the resistance of PrAg-PCIS to cleavage by endogenous furin-like proteases (Figure 1B). Incubation of the cells with the furin-activatable PrAg-WT results in the rapid processing of 83-kDa PrAg-WT to the activated 63-kDa form within 15 minutes, and by 6 hours, all of the PrAg-WT was processed to the PrAg-WT 63-kDa form in both HEK/GPI-testisin and HEK/vector cells (Figure 4B). Loss of the 83-kDa PrAg-WT occurred more rapidly in HEK/GPI-testisin cells, possibly reflecting increased processing due to the presence of testisin, in addition to furin.

### PrAg-PCIS toxin is cytotoxic to cells expressing active GPI-anchored testisin

To investigate potential tumor cell killing resulting from testisin activation of PrAg-PCIS, cytotoxicity assays were performed using HEK/GPI-testisin and HEK/vector cells. HEK/GPI-testisin cells showed a dose-dependent sensitivity to killing by PrAg-PCIS toxin (Figure 4C), similar to the furin-dependent PrAg-WT toxin (EC_50_ 3 ng/mL for PrAg-PCIS *vs* 3 ng/mL for PrAg-WT) (Figures 4C, 4D). HEK/vector cells were 10-fold less sensitive to PrAg-PCIS toxin (EC_50_ 30 ng/mL), while showing similar susceptibility to the furin-dependent PrAg-WT toxin (EC_50_ 3 ng/mL) (Figures 4C, 4D). FP59 and the PrAg proteins did not cause cellular cytotoxicity when incubated with the cells individually (data not shown). These data show that testisin can increase PrAg-PCIS activation and toxin-induced cytotoxicity. The dependence of this activity on active testisin was examined using HEK293T cells stably expressing two catalytically inactive testisin mutants, R41A-testisin and S238A-testisin (Supplementary Figure S2). The R41A-testisin mutant encodes an Ala for Arg^41^ mutation in the activation site of the testisin zymogen, thus maintaining the enzyme in a ‘zymogen locked,’ inactive conformation [75]. When HEK293T cells expressing R41A-testisin were incubated with the PrAg-PCIS toxin, viability was similar to that seen in the HEK/vector cell line (EC_50_ 30 ng/mL for HEK/R41A-testisin *vs* 30 ng/mL for HEK/vector) (Figure 4C). The S238A-testisin mutant encodes a substitution of Ala for Ser^238^ of the catalytic triad, which is required for the mechanism of peptide bond cleavage by serine proteases [76]. Detection of the S238A-testisin mutant when expressed in HEK293T cells was relatively poor when compared with detection of the R41A-testisin mutant or testisin in these cells (Supplementary Figure S2) for unknown reasons. When incubated with PrAg-PCIS toxin, the presence of S238A-testisin did not result in increased activation of PrAg-PCIS toxin, as viability of the HEK/S238A-testisin cells was similar to that of the HEK/R41A-testisin and HEK/vector alone cell lines (EC_50_ 30 ng/mL) (Figure 4C). As expected, cells expressing S238A-testisin and R41A-testisin mutants were as susceptible to killing by the furin-dependent PrAg-WT toxin as the HEK/GPI-testisin cells (EC_50_ 3 ng/mL for HEK/S238A-testisin; EC_50_ 3 ng/mL for HEK/R41A-testisin) (Figure 4D). Together, these data show that testisin activity is responsible for the increased PrAg-PCIS induced cytotoxicity in HEK/GPI-testisin cells.

### Tumor cells expressing endogenous testisin are killed by the PrAg-PCIS toxin

**Figure 5:**
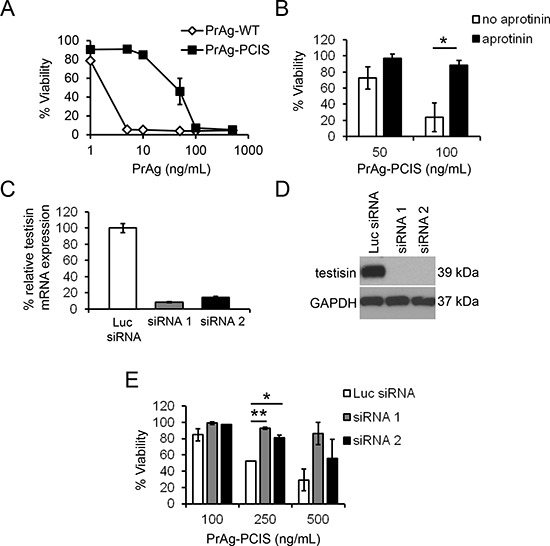
Endogenous testisin activity activates the PrAg-PCIS toxin and promotes HeLa tumor cell killing **A.** HeLa cells are sensitive to the PrAg-PCIS toxin. HeLa cells were incubated with 0–500 ng/mL of PrAg proteins (PrAg-PCIS or PrAg-WT) and FP59 (50 ng/mL) for 48 hours and then assayed for cell viability by MTT assay. Values are calculated from two independent experiments performed in triplicate **B.** Aprotinin-sensitive proteases contribute to PrAg-PCIS toxin-induced cytotoxicity. HeLa cells were pre-incubated in the presence of a final concentration of 100 μM aprotinin for 30 minutes, prior to treatment with the indicated concentrations of PrAg-PCIS and FP59 (50 ng/mL) for 2 hours. Media was replaced and cell viability assayed 48 hours later by MTT assay. Values are calculated from two independent experiments performed in triplicate. *p < 0.05. **C.** siRNA knockdown of testisin mRNA expression in HeLa cells. mRNA expression levels are normalized to GAPDH and expressed relative to the Luc-siRNA control. **D.** Immunoblot analysis of testisin protein expression after siRNA knockdown. The blot was probed using anti-testisin antibody and reprobed with anti-GAPDH antibody. Data is representative of at least two independent experiments. **E.** Depletion of testisin reduces the sensitivity of HeLa cells to PrAg-PCIS toxin-induced cytotoxicity. Testisin siRNA or control Luc-siRNA transfected HeLa cells were incubated for 6 hours with indicated concentrations of PrAg-PCIS and FP59 (50 ng/mL). Media was replaced and cell viability was assayed 48 hours later by MTT assay. Values are the means calculated from two independent experiments performed in triplicate. *p < 0.05; **p < 0.01.

To investigate the activation of PrAg-PCIS toxin by endogenous testisin in a natural tumor cell system, HeLa cervical cancer cells, which constitutively express testisin [63, 77], were treated with the PrAg-PCIS toxin. Increasing concentrations of PrAg-PCIS toxin resulted in substantial HeLa cell death that was dose-dependent, although HeLa cells were less sensitive to the PrAg-PCIS toxin than to the PrAg-WT toxin (Figure 5A). The FP59 and the PrAg proteins did not induce cytotoxicity when incubated with the cells individually (data not shown). Pre-incubation of the HeLa cells with aprotinin, which has been shown to inhibit testisin activity [77], prior to the addition of the PrAg-PCIS toxin, resulted in significant attenuation of toxicity (Figure 5B), demonstrating that PrAg-PCIS toxin-induced cytotoxicity in HeLa cells is dependent on cell-surface serine protease activity, and suggesting that testisin may contribute to PrAg-PCIS activation on HeLa cells. The specific dependence of PrAg-PCIS toxin-induced cytotoxicity on the presence of testisin was revealed following knockdown of testisin expression in HeLa cells using siRNA. Efficient knockdown of testisin mRNA (Figure 5C) and protein (Figure 5D) levels were achieved using two independent testisin-specific siRNAs, compared to a control siRNA (Luc-siRNA). Incubation of the siRNA control cells with increasing concentrations of PrAg-PCIS toxin produced a dose-dependent decrease in cell viability, whereas HeLa cells depleted of testisin were relatively resistant to killing by the PrAg-PCIS toxin (Figure 5E). Together, these data demonstrate that testisin is a significant contributor to PrAg-PCIS toxin activation on HeLa cells.

### PrAg-PCIS toxin is cytotoxic to tumor cells expressing active hepsin, but not matriptase

**Figure 6:**
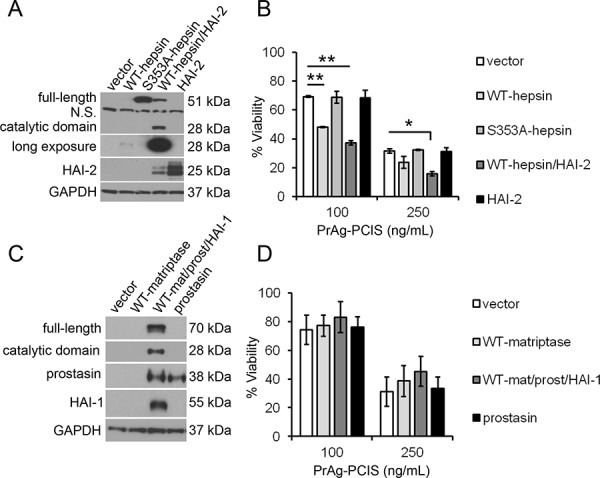
Cellular hepsin is an activator of PrAg-PCIS toxin on tumor cells **A.** Detection of hepsin expressed in HeLa cells. HeLa cells were transfected with full-length hepsin (WT-hepsin), an inactive hepsin catalytic mutant (S353A-hepsin), WT-hepsin and HAI-2, HAI-2, or vector alone. After 48 hours, lysates were analyzed by immunoblot and probed using anti-hepsin, anti-HAI-2, and anti-GAPDH antibodies. The 28-kDa hepsin catalytic domain, detected under reducing conditions, is a product of activation of the 51-kDa hepsin zymogen and is a measure of the presence of active hepsin. The long exposure allows detection of the low levels of active hepsin in the absence of HAI-2. The blot is representative of at least two independent experiments. **B.** Hepsin expression in HeLa cells enhances PrAg-PCIS toxin-induced cytotoxicity. Control and hepsin expressing HeLa cells were incubated with indicated concentrations of PrAg-PCIS and FP59 (50 ng/mL) for 6 hours. Media was then replaced and cell viability assayed after 24 hours by MTT assay. Values are the means calculated from two independent experiments performed in triplicate. *p < 0.05; **p < 0.01. **C.** Detection of matriptase expressed in HeLa cells. HeLa cells were transfected with full-length matriptase (WT-matriptase), prostasin, vector alone, or were co-transfected with matriptase, prostasin, and HAI-1. After 48 hours, lysates were analyzed by immunoblot using anti-matriptase, anti-prostasin, anti-HAI-1, and anti-GAPDH antibodies. The 28-kDa matriptase catalytic domain detected under reducing conditions is evidence of active matriptase produced upon activation of the 70-kDa zymogen form of matriptase. The blot is representative of at least two independent experiments. **D.** Matriptase expression in HeLa cells does not enhance PrAg-PCIS toxin-induced cytotoxicity. Control and matriptase expressing HeLa cells were incubated with indicated concentrations of PrAg-PCIS and FP59 (50 ng/mL) for 6 hours. Media was then replaced and cell viability assayed after 24 hours by MTT assay. Values are the means calculated from two independent experiments performed in triplicate.

The activation cleavage of PrAg-PCIS by both recombinant matriptase and hepsin *in vitro* suggested that the full-length forms of these membrane-tethered enzymes could be additional activators of PrAg-PCIS. To test the role of cell-expressed hepsin in activating PrAg-PCIS, HeLa cells were transfected with expression plasmids encoding full-length hepsin or an inactive S353A-hepsin catalytic mutant (Figure 6A). Because transfection of full-length hepsin results in low levels of detectable hepsin protein (Figure 6A), hepatocyte growth factor activator inhibitor-2 (HAI-2, *SPINT2*), which likely functions as a chaperone protein to enhance hepsin protein stability, was also co-expressed with hepsin (Figure 6A). The expression of hepsin in HeLa cells produced active hepsin, evidenced by the presence of a 28-kDa hepsin catalytic domain, which is produced after activation cleavage of the hepsin zymogen. The presence of full-length hepsin alone resulted in a 30% increase in PrAg-PCIS toxin-induced cytotoxicity in HeLa cells, and the HAI-2-enhanced hepsin activity resulted in a 43% increase in toxin-induced cytotoxicity relative to control cells (Figure 6B), suggesting that cell surface hepsin is an activator of PrAg-PCIS.

To test the role of cell-expressed matriptase in activating PrAg-PCIS, full-length matriptase was expressed in HeLa cells. Efficient matriptase expression required co-expression with hepatocyte growth factor activator inhibitor-1 (HAI-1, *SPINT1*) and prostasin (*PRSS8*), to enhance matriptase trafficking to the cell surface [78, 79] and increase matriptase zymogen activation [80, 81] (Figure 6C). Co-expression of matriptase, HAI-1, and prostasin generated active matriptase as evidenced by the presence of the 28-kDa matriptase catalytic domain, which is produced after activation cleavage of the matriptase zymogen [82] (Figure 6C). In contrast to hepsin, PrAg-PCIS activation and toxin-induced cytotoxicity was unaffected by the presence of matriptase (Figure 6D). These data show that although the catalytic domain of matriptase is capable of PrAg-PCIS activation in solution, matriptase may not be a major contributor to PrAg-PCIS toxin activation on the cell surface, whereas hepsin likely contributes to PrAg-PCIS toxin activation on tumor cells that express hepsin.

### PrAg-PCIS toxin inhibits tumor growth in a preclinical xenograft mouse model

**Figure 7:**
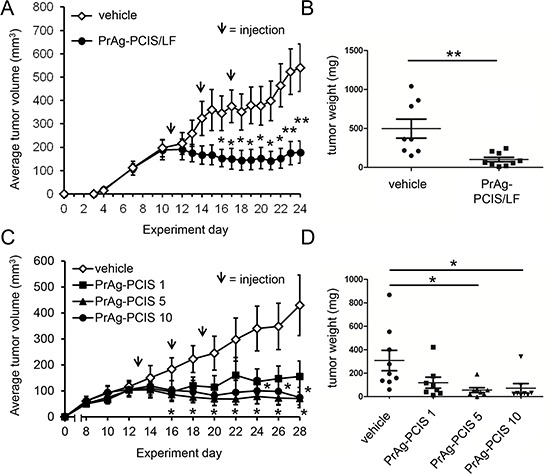
PrAg-PCIS toxin is a potent cytotoxic agent for HeLa tumor xenografts **A.** Treatment with PrAg-PCIS toxin inhibits growth of subcutaneous HeLa xenograft tumors in nude mice. Average tumor volumes measured for HeLa tumors injected with 10 μg PrAg-PCIS combined with 5 μg LF or vehicle (PBS alone) on day 11, day 14, and day 17 (indicated by arrows) after inoculation of HeLa cells (day 0). Mice: *n* = 8 vehicle; *n* = 9 PrAg-PCIS/LF. **B.** Tumor weights obtained after resection of tumors in A). **C.** Dose dependence of PrAg-PCIS toxin in subcutaneous HeLa xenograft tumors. Average tumor volumes measured for HeLa tumors injected with 1 μg PrAg-PCIS, 5 μg PrAg-PCIS, 10 μg PrAg-PCIS, or vehicle (PBS combined with 5 μg LF) on day 13, day 16, and day 19 (indicated by arrows) after inoculation of HeLa cells (day 0). Mice: *n* = 9 vehicle; *n* = 8 for each of PrAg-PCIS 1 μg, PrAg-PCIS 5 μg, and PrAg-PCIS 10 μg. **D.** Tumor weights obtained after resection of tumors in C). *p < 0.05, **p < 0.01.

The ability of the PrAg-PCIS toxin to inhibit tumor growth *in vivo* was examined using a xenograft mouse model. Athymic female nude mice bearing subcutaneous HeLa tumors received three intratumoral injections (one every three days) of PrAg-PCIS toxin (10 μg PrAg-PCIS and 5 μg LF) or vehicle alone (PBS), and tumor growth was assessed by caliper measurements. LF was used *in vivo* in place of FP59 to avoid any off-target effects that may be associated with non-specific uptake of the very effective protein translation inhibitor FP59 [27]. After the first injection of PrAg-PCIS toxin, tumor growth arrested and did not increase compared with vehicle treated tumors, over the course of the experiment (Figure 7A). Tumors were harvested and weighed up to 7 days after the final treatment. Tumor weights correlated well with measures of tumor volumes, with the mouse cohort that received PrAg-PCIS toxin showing a significant 5-fold reduction in average tumor weight relative to the cohort treated with vehicle alone (Figure 7B).

The dose-dependence of tumor growth inhibition by PrAg-PCIS toxin was also investigated using this xenograft model. Cohorts of mice bearing subcutaneous HeLa tumors received three injections (one every three days) composed of 10 μg, 5 μg, 1 μg PrAg-PCIS toxin, or vehicle (5 μg LF in PBS). Tumor growth as assessed by caliper measurements again showed tumor growth arrest in all 3 cohorts treated with PrAg-PCIS toxin compared with vehicle alone treated animals over the course of the experiment (Figure 7C). The tumor weights obtained at the end of the experiment correlated well with the measured tumor volumes (Figures 7C, 7D). The tumor volumes measured in mice treated with 10 μg and 5 μg doses of PrAg-PCIS toxin decreased significantly over the course of the experiment, showing 4.3-fold and 5.6-fold reduced average tumor weights, respectively, compared to vehicle alone, at the end of the experiment (Figures 7C, 7D). Tumors treated with the 1 μg dose of PrAg-PCIS toxin showed a non-significant trend toward reduced average tumor volume and average tumor weight relative to mice treated with vehicle alone (Figures 7C, 7D). Treatments with the PrAg-PCIS toxin were well-tolerated by the mice and did not appear to have any overt off-target side effects. Treated mice did not experience substantial weight loss (Supplementary Figure S3) and necropsies revealed no gross abnormalities or organ damage (data not shown). These data demonstrate a significant effect of the PrAg-PCIS toxin in inhibiting tumor growth in a preclinical mouse model.

**Figure 8:**
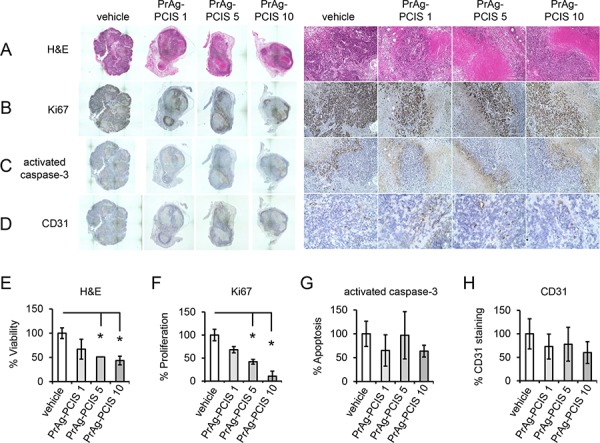
PrAg-PCIS toxin treatment increases tumor necrosis and reduces tumor cell proliferation **A–D.** Histology and immunohistochemical analyses performed on serial sections of tumors resected from mice treated with PrAg-PCIS 1 μg, PrAg-PCIS 5 μg, and PrAg-PCIS 10 μg or vehicle alone (PBS/LF). Representative serial sections and high power magnified fields are shown to reveal gross tumor morphology, overall tumor staining, and regions of necrosis and proliferation, as well as antibody specificity. **E–H.** Composite images compiled from each stained section were analyzed to determine % tumor viability (H&E), % tumor cell proliferation (Ki67), % apoptosis (activated caspase-3), and % vessel density (CD31), as indicated. Tumors: *n* = 4 vehicle; *n* = 3 PrAg-PCIS 1 μg; *n* = 2 PrAg-PCIS 5 μg; *n* = 3 PrAg-PCIS 10 μg. *p < 0.05.

Quantitative histomorphometric analyses were performed on serial sections of the harvested tumors to investigate the mechanistic basis for the potent anti-tumor activity of the PrAg-PCIS toxin. Microscopic analysis of sections stained with hematoxylin/eosin (H&E) showed that tumors exposed to either 10 μg PrAg-PCIS toxin or 5 μg PrAg-PCIS toxin presented with substantial areas of necrosis, as indicated by reduced staining of the tissue and the presence of patches of destroyed tumor with loss of nuclei (Figures 8A, 8E), which was not seen in the vehicle treated control group, which had significantly more viable tumor area (increased approximately 2-fold relative to toxin treated groups) (Figures 8A, 8E). The tumors treated with 1 μg PrAg-PCIS toxin also showed reduced staining and loss of viability, which did not quite reach statistical significance relative to the vehicle treated control group (Figures 8A, 8E). Staining for the proliferation marker Ki67 revealed that tumor cell proliferation in tumors treated with 10 μg PrAg-PCIS toxin or 5 μg PrAg-PCIS toxin was significantly reduced by 3.3-fold and 2.3-fold respectively, relative to vehicle treatment, and was associated only with the remaining viable areas of the tumors (Figures 8B, 8F). Apoptotic cells, evidenced by staining for activated caspase-3, were concentrated in the areas peripheral to the necrotic areas and adjacent to the viable areas of the tumors, but overall differences were not observed amongst the treatment groups (Figures 8C, 8G). Likewise, vessel density, as measured by CD31 staining, appeared not to be significantly affected by PrAg-PCIS toxin treatment and staining of vessels was confined to the viable areas of the tumors (Figures 8D, 8H). This data suggests that PrAg-PCIS toxin treatment inhibits tumor growth through the reduction of tumor cell proliferation and the induction of tumor necrosis.

## DISCUSSION

The enzymatic activities of proteases are frequently elevated in tumors and in the tumor microenvironment due to increased tumor cell expression of proteases and disruption to the regulatory networks responsible for tightly regulating their functions. In the present study, we sought to determine whether the tumor-associated membrane-anchored serine protease testisin could be targeted for anti-tumor therapies. We took advantage of the requirement for anthrax toxin to be proteolytically activated on the cell surface to engineer a novel testisin-activated anthrax toxin, PrAg-PCIS toxin. We established that the PrAg-PCIS toxin can be activated by tumor cell-expressed testisin and inhibit the growth of tumor cells in both cell culture and in testisin-expressing tumors *in vivo*.

We found that the engineered PrAg-PCIS toxin was resistant to activation by furin, and was able to be activated by both recombinant mouse testisin and cell-expressed human testisin. Activation by testisin was strictly dependent upon testisin's catalytic activity, as the inactive mutant testisin proteins could not activate PrAg-PCIS toxin. In addition to demonstrating that PrAg-PCIS toxin can be activated by tumor-expressed testisin to inhibit the growth of tumor cells in culture, we also demonstrated that it can inhibit the growth of a testisin-expressing tumor cell line *in vivo*, and it was well-tolerated by the mice with no obvious adverse reactions. The growth inhibitory effect of the PrAg-PCIS toxin may be explained by the inhibition of tumor cell proliferation and induction of necrosis. Other engineered anthrax toxins have been shown to kill tumor cells by inducing tumor necrosis [18, 21], increasing tumor cell apoptosis [27], as well as targeting tumor-associated vasculature [18, 19, 24]. We did not detect a significant decrease in vessel density within tumors by CD31 staining, or an increase in tumor cell apoptosis as measured by the presence of activated caspase-3. The retention of these latter tumor properties was notable given the striking decrease in tumor mass after treatment with the PrAg-PCIS toxin.

Although the engineered cleavage site in PrAg-PCIS showed a preference for activation cleavage by testisin, it was also able to be cleaved *in vitro* by the recombinant catalytic domains of additional serine proteases, notably hepsin and matriptase, suggesting that activation of PrAg-PCIS is not completely specific to testisin, but that additional active pericellular serine proteases overexpressed by tumor cells may also be effectively targeted by this modified anthrax toxin prodrug strategy. As hepsin, and multiple other membrane-anchored serine proteases have been found to be overexpressed in multiple tumor types, this may be an unexpected beneficial feature of PrAg-PCIS. The PrAg-PCIS toxin prodrug may be useful in targeting multiple overexpressed tumor antigen proteases, rather than a single protease. The activation of PrAg-PCIS toxin by testisin and hepsin expressed by tumor cells in cell culture reflected the cleavage of PrAg-PCIS *in vitro*, yet matriptase was ineffective at activating PrAg-PCIS toxin when expressed as a full-length protein on the cell surface. Thus, the protease cleavage reactions observed *in vitro* may not entirely reflect the *in vivo* targets. The reason for lack of effective full-length matriptase-mediated PrAg-PCIS toxin activation on the surface of tumor cells is not known, but could be due to an inability to access the PrAg-PCIS cleavage site due to steric hindrance, or due to other factors preventing activation of PrAg-PCIS by matriptase.

Anthrax toxin has many features that make it optimal for engineering into a protease-activated tumor-targeted cytotoxin. The primary anthrax toxin receptor, CMG2, is expressed in nearly all tissues [83], allowing for the targeting of tumors of diverse tissue origin. Moreover, previous studies have revealed that anthrax toxin can be engineered to target distinct anthrax toxin receptors [25] or alternative receptors such as EGFR (Epidermal Growth Factor Receptor-1, *ERBB1*) [84] and HER2 (Human Epidermal Growth Factor Receptor-2, *ERBB2*) [85], whose presence is enriched on the surfaces of certain tumors. Additionally, the requirement for anthrax toxin to be proteolytically activated has been the basis of engineering anthrax toxin to be activated by proteases that are overexpressed and hyperactive on the surfaces of tumor cells. These properties of engineered anthrax toxins can be considered to impart a degree of tumor specificity not provided by classic protease inhibition, which may limit off-target effects of engineered anthrax toxins used as tumor therapies. Engineered anthrax toxins also function as highly efficient protein delivery systems, capable of delivering modified payloads into cells to induce cytotoxic or potentially other effects. When combined with engineered PrAgs, both LF (as an anti-tumor cell and anti-angiogenic drug) and FP59 have been used successfully as anti-tumor therapies in preclinical studies. It is foreseeable that future payloads may be rationally designed to exert cytotoxic or signal-system modulator effects specifically in the cytosol of tumor cells, while remaining harmless in the cytosol of non-transformed cells.

To our knowledge, this is the first study in which a member of the membrane-anchored serine protease family has been targeted with a protease-activated prodrug-like reagent. The finding that testisin and hepsin enzymatic activities can be targeted on tumor cells by PrAg-PCIS suggests that the membrane-anchored serine proteases constitute viable targets for engineered anthrax toxins. Additionally, these studies suggest that other prodrug approaches, such as use of other protease-activated toxins [86–88] or activatable cell penetrating peptides (ACPPs) [89–91], may be adapted to target the proteolytic activities of tumor-expressed membrane-anchored serine proteases for therapeutic or diagnostic purposes.

## MATERIALS AND METHODS

### Reagents

Enzymes for recombinant DNA preparation were purchased from New England BioLabs. Recombinant mouse testisin (6820-SE-10), human hepsin (4776-SE-10), human prostasin (4599-SE), and HAT (2695-SE) were purchased from R&D Systems. Each protease was activated according to the manufacturer's instructions. Recombinant human thrombin (470HT) and recombinant human uPA (ADG125N) were purchased from American Diagnostica. Recombinant human PCI and mouse anti-PCI antibody were prepared as previously described [92, 93]. Briefly, recombinant PCI was prepared in *Escherichia coli* and purified using Ni^2+^-chelate and heparin-sepharose affinity chromatography, as in [93]. Recombinant human furin was provided by Dr. Iris Lindberg (University of Maryland School of Medicine, Baltimore, MD) [94]. Recombinant human matriptase was provided by Dr. Richard Leduc (Universite de Sherbrooke, Quebec, Canada) [95]. Human aPC was provided by Dr. Li Zhang (University of Maryland Baltimore School of Medicine, Baltimore, MD) [96]. Aprotinin (A1153) was purchased from Sigma-Aldrich. Rabbit anti-PrAg antibody (no. 5308) was prepared as previously described [16]. Additional antibodies included goat anti-HAI-1 (AF1048) and goat anti-HAI-2 (AF1106) (R&D Systems), rabbit anti-matriptase (IM1014) (Calbiochem), mouse anti-prostasin (612172) (BD Transduction Laboratories), rabbit anti-glyceraldehyde 3-phosphate dehydrogenase (GAPDH) (14C10) (Cell Signaling Technologies), rabbit anti-hepsin (100022) (Cayman Chemical); anti-mouse and anti-rabbit horseradish peroxidase (HRP)-conjugated antibodies (Jackson ImmunoResearch Laboratories), and anti-goat HRP-conjugated antibody (KPL). Mouse Pro1.4.C25.1 anti-testisin antibody was produced by standard procedures from a hybridoma cell line (PTA-6076) (ATCC).

### Real-time quantitative PCR (qPCR)

RNA was isolated from cell lines using the RNeasy Kit (Qiagen). Reverse transcription was performed using Taqman Reverse Transcription Reagents (Applied Biosystems). qPCR was performed using testisin (Hs00199035_m1), hepsin (Hs01056332_m1), matriptase (Hs00222707_m1), GAPDH (Hs02758991_g1) and beta-actin (β-actin) (Hs99999903) primers and Taqman RT-PCR reagents (Applied Biosystems). mRNA expression levels were normalized to GAPDH or β-actin.

### Cell lysis and immunoblotting

Cells were lysed in cell lysis buffer (150 mM NaCl, 10 mM CaCl_2_, 50 mM HEPES (pH 7.3), 0.5% Triton X-100, 0.5% NP-40, Complete Mini-EDTA Protease Inhibitor Cocktail (Roche)), and protein concentrations determined by Bradford assay. Samples containing equal protein were heated at 95°C for 5 minutes in Laemmli sample buffer containing 10% beta-mercaptoethanol and analyzed by SDS-polyacrylamide gel electrophoresis (PAGE), using 4–12% or 10% NuPage Bis-Tris pre-cast gels (Life Technologies), followed by immunoblotting using PVDF membranes (Life Technologies). Membranes were blocked for 30 minutes in 5% (w/v) non-fat milk and then sequentially incubated with primary and HRP-conjugated secondary antibodies. HRP activity was detected using SuperSignal West Pico Chemiluminescent Substrate (Thermo Scientific).

### Plasmids and mutagenesis

A two-step overlap PCR strategy was employed to mutate the cDNA bases encoding the furin cleavage site in the PrAg expression plasmid pYS5-PA33 [97]. pYS5-PA33 served as the template for the first round of PCR using the primers denoted ‘A’ (below). The resulting PCR reaction was digested with DpnI and the mutant plasmid cloned by standard techniques and used as the template for the second round of PCR using primers denoted 'B’ (below). The resulting PCR reaction was digested with DpnI and the final mutant plasmid cloned and verified by DNA sequencing.

PrAg-PCIS ‘A’:

F:5′GCTGCTAGATCGGCGCGTCTAGGACCTACGG3′

R:5′CCGTAGGTCCTAGACGCGCCGATCTAGCAGC3′

PrAg-PCIS ‘B’:

F:5′CTTCGAATTCATTCACGTTTAGATCGGCGCGTCTAGG3′

R:5′CCTAGACGCGCCGATCTAAACGTGAATGAATTCGAAG3′

Expression plasmids encoding human matriptase [98], human HAI-1 [98], and human HAI-2 [99] were provided by Dr. Chen-Yong Lin (Georgetown University, Washington DC). cDNA encoding human testisin (GPI-testisin) [54], cloned into pcDNA3.1 expression plasmid (Life Technologies), was mutated by site-directed mutagenesis using the primers denoted below using the QuikChange Mutagenesis kit (Stratagene) to create ‘zymogen-locked’ activation site (R41A-testisin) and catalytic triad (S238A-testisin) mutants of testisin. Similarly, cDNA encoding human hepsin (WT-hepsin) [100], cloned into pcDNA 3.1, was mutated to create a catalytic triad S353A-hepsin mutant (S353A-hepsin). Cloning and mutagenesis accuracy was verified by DNA sequencing.

R41A-testisin:

F:5′GGGTCATCACGTCGGCGATCGTGGGTGG3′

R:5′CCTCTCCACCCACGATCGCCGACGT3′

S238A-testisin:

F:5′CCTGCTTCGGTGACGCAGGCGGACCCTTGG3′

R:5′CAGGCCAAGGGTCCGCCTGCGTCAC3′

S353A-hepsin:

F:5′GCCTGCCAGGGCGACGCGGGTGGTCCCTTTGTG3′

R:5′CACAAAGGGACCACCCGCGTCGCCCTGGCAGGC3′

### Expression and purification of PrAg proteins

Recombinant anthrax toxin protective antigens (PrAg-WT, PrAg-PCIS), recombinant LF, and FP59 were generated and purified as previously described [16, 69]. Briefly, expression plasmids containing PrAg sequences contained in the *E. coli-Bacillus* expression plasmids pYS5 or pYS5-PA33, were transformed into the non-virulent *B. anthracis strain* BH460. The proteins were secreted into the culture supernatants and purified by ammonium sulfate precipitation and chromatography on a Mono-Q column to high yield and purity, as described [69]. The LF and FP59 used herein have the native N-terminal sequence of AGG [101].

### PCI cleavage assay

Recombinant hepsin or matriptase (50 nM) were incubated with 50 nM recombinant PCI. Recombinant testisin (50 nM) was incubated with 500 nM recombinant PCI. After 30 minutes of incubation at room temperature, in 50 mM Tris-HCl (pH 7.5), 150 mM NaCl, and 10 mM CaCl_2_, or at indicated intervals, Laemmli sample buffer containing 10% beta-mercaptoethanol was added to the reactions. Samples were immunoblotted for PCI cleavage or protease-PCI complex formation using anti-PCI, anti-hepsin, or anti-matriptase antibodies.

### Peptide assays

Peptide cleavage assays were performed using 10 nM recombinant testisin, 10 nM recombinant hepsin, or 100 nM recombinant matriptase, and 100 nM chromogenic succinyl-AAPR-*p*-nitroaniline peptide (Bachem). Reaction absorbance (abs) values were measured at 420 nm using a spectrophotometer (TECAN) at times indicated in the figure legend. The change in absorbance units is relative to the absorbance measured in the absence of peptide substrate. The absorbance of peptide substrate alone did not increase in the absence of protease over time.

### *In vitro* PrAg cleavage assays

Recombinant PrAg proteins (1 μM) were incubated with recombinant proteases (50 nM) for 2.5 hours, or indicated intervals, at 30°C, in 50 mM HEPES (pH 7.3), 10 mM CaCl_2_, 150 mM NaCl, and 0.05% (v/v) Brij-35. Reactions were stopped by addition of Laemmli sample buffer containing 10% beta-mercaptoethanol to the samples. PrAg cleavage was analyzed by SDS-PAGE followed by immunoblotting using anti-PrAg antibody.

For densitometry of PrAg processing, all values were measured using Image J software and normalized to GAPDH expression. Individual PrAg 83-kDa and 63-kDa values for each timepoint are calculated relative to the sum value of PrAg 83-kDa and 63-kDa at that timepoint, which was set equal to 1.

### Cell culture and transfections

Human cell lines were purchased from American Type Culture Collection (ATCC), with the exception of NCI/ADR-Res cells, which were purchased from the NCI-DCTD repository (Frederick, MD). Cell lines were cultured and maintained at 37°C in a 5% CO_2_/95% air environment in Dulbecco's Modified Eagle's Medium (DMEM) supplemented with 10% heat-inactivated fetal bovine serum (FBS) and 100 units/mL penicillin, 100 μg/mL streptomycin, and 2 mM L-glutamine. All cells were routinely tested and confirmed to be free of mycoplasma contamination. HEK293T cells were transfected with expression plasmids encoding full-length human GPI-anchored testisin (HEK/GPI-testisin), S238A-testisin catalytic triad mutant (HEK/S238A-testisin), R41A-testisin ‘zymogen-locked’ activation site mutant (HEK/R41A-testisin), or vector alone (HEK/vector) using Lipofectamine 2000 (Life Technologies). Two stably-transfected pools of each transfection were obtained by selection in hygromycin and testisin/mutant expression determined by immunoblot (Supplementary Figure S2). HeLa cells were transiently transfected or co-transfected with expression plasmids encoding matriptase (WT-matriptase), prostasin [102], hepsin (WT-hepsin), S353A-hepsin catalytic triad mutant (S353A-hepsin), HAI-1, HAI-2, or vector alone (vector) using Lipofectamine 2000.

### Knockdown by RNA interference

HeLa cells were transfected with 20 nM testisin-specific STEALTH siRNAs (HSS116894; HSS173992) (Life Technologies) or 20 nM luciferase-specific negative control (Luc-siRNA) (Life Technologies) using Dharmafect 1 (Dharmacon). After 48 hours, cells were harvested for analysis of testisin mRNA and protein expression, or used in MTT cytotoxicity assays. The efficiency of testisin knockdown was analyzed by qPCR and immunoblotting.

### MTT cytotoxicity assays

Cells were incubated with various concentrations of PrAg-PCIS or PrAg-WT (as indicated in figure legends) and FP59 (50 ng/mL) in growth media for indicated times. Media was replaced with fresh media and cell viability was assayed from 24–48 hours later (as indicated in the figure legends) by adding MTT (3-(4,5-dimethylthiazol-2-yl)-2,5-diphenyltetrazolium bromide) (Millipore) to a final concentration of 1.25 mg/mL, and incubating for 45 minutes to one hour at 37°C. MTT was dissolved in growth media and filtered through a 0.22 μm syringe filter. The formed pigment was solubilized with 0.5% (w/v) SDS, 25 mM HCl, in 90% (v/v) isopropanol. Absorbance was measured using a spectrophotometer (TECAN) at 550 nm and 620 nm (reference wavelength). Values obtained for incubation of cells with PrAg toxins were normalized to those obtained for the cells incubated with FP59 alone (100%). EC_50_ is defined as the concentration (derived from the viability plots) of PrAg toxin required to kill 50% of the cells.

### *In vivo* tumor xenograft models

Female athymic nude mice (NU/NU) (6–8 wks old) (Charles River) were housed and monitored according to Institutional Animal Care and Use Committee guidelines, given free access to food and water, and maintained in a 12 hour dark/light environment. 2.5 × 10^6^ HeLa tumor cells were injected subcutaneously into the right hind flanks of the mice. Upon measurable tumor growth (~50–200 mm^3^), mice were distributed into cohorts containing mice bearing approximately equal individual tumor volumes and approximately equal average tumor volumes. Each mouse received a 100 μL intratumoral injection, injected into multiple spots in the tumor, every three days for a total of three injections. Tumor dimensions were measured with calipers at indicated timepoints in a blinded manner with respect to tumor treatment. Tumor volume was calculated using the formula 0.5 × length × width^2^. Experiments were concluded when one or more mice reached predetermined endpoints (weight gain > 10%, tumor diameter > 1 cm, tumor ulceration). Mice were then euthanized and tumors were removed, weighed (in a treatment-blinded manner), fixed in 10% zinc buffer, and stored in 70% ethanol for histology and immunohistochemical analysis.

### Histopathological analysis

Zinc-fixed tumor specimens were embedded in paraffin and 5 μm-thick sections were cut, deparafinized, and stained with hematoxylin and eosin (H&E) using standard procedures, or subjected to immunohistochemical analysis. For immunohistochemistry, samples were rehydrated, endogenous peroxidase activity blocked with 3% hydrogen peroxide in methanol, subjected to antigen retrieval in boiling sodium citrate, and then non-specific binding sites blocked with 5% goat serum. Sections were incubated overnight at 4°C with 1:100 dilutions of rabbit anti-Ki67 (ab16667) (Abcam), rabbit anti-human activated caspase-3 (9661S) (Cell Signaling Technology), or rat anti-mouse CD31 (553370) (BD Pharmingen), followed by incubation for 30 minutes with 1:200 anti-rat or anti-rabbit biotinylated secondary antibodies. Antibody binding was detected using a Vectastain ABC Kit (Vector Laboratories). Sections were counterstained with hematoxylin, dehydrated, and mounted. Control slides were incubated with primary or secondary antibodies only. Images were obtained using an EVOS FL Auto Cell Imaging System (Life Technologies). Composite images of the whole tumor sections were obtained with a 10x objective and stitched together using the EVOS software, while individual fields were taken using 20x (H&E, Ki67, activated caspase-3) or 40x (CD31) objectives, respectively. Staining was quantified using Image J software (H&E, Ki67, activated caspase-3) and Photoshop (Adobe) (CD31). Quantification, performed in a treatment-blinded manner, was performed by outlining the tumors in the composite images and analyzing the tumor sections for % viable area (H&E) or % positive staining for the immunostained sections. Percentages were calculated using the ratio of viable area or stained area of the tumor to the total tumor area (areas determined by pixel count), as described in [103].

### Statistical analysis

Quantitative data are represented as mean values with their respective standard errors (SEM). Significance (relative to vector or vehicle control groups) was tested using unpaired two-tailed Student's *t* test, which was calculated using GraphPad software. *p* values < 0.05 were considered statistically significant.

## SUPPLEMENTARY FIGURES


