# Supplementary material for: Targeting the membrane-anchored serine protease testisin with a novel engineered anthrax toxin prodrug to kill tumor cells and reduce tumor burden
Source: Oncotarget. 2015 Sep 15;6(32):33534–53. doi: 10.18632/oncotarget.5214 (PMC4741784; doi:10.18632/oncotarget.5214)
Supplement: Supplementary file 1 [file oncotarget-06-33534-s001.pdf]

SUPPLEMENTARY FIGURES

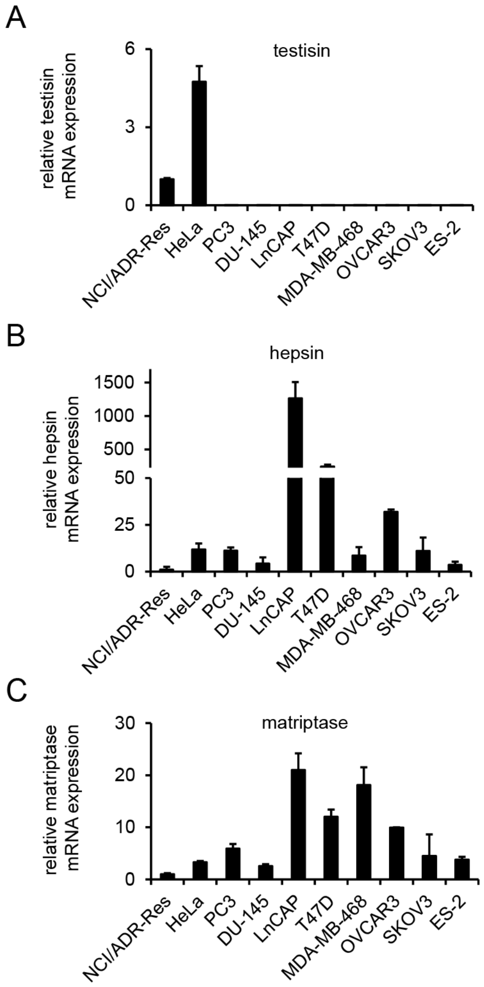

Supplementary Figure S1: Expression of testisin, hepsin, and matriptase in tumor cell lines. Tumor cell lines that were treated with PrAg-PCIS toxin (Figure 1) were subject to qPCR to measure relative mRNA levels of **A.** testisin, **B.** hepsin, and **C.** matriptase. mRNA is normalized to β-actin and expressed relative to the mRNA levels detected in NCI/ADR-Res cells. The results reveal that all of the cells expressed mRNA encoding one or more of the proteases.

w

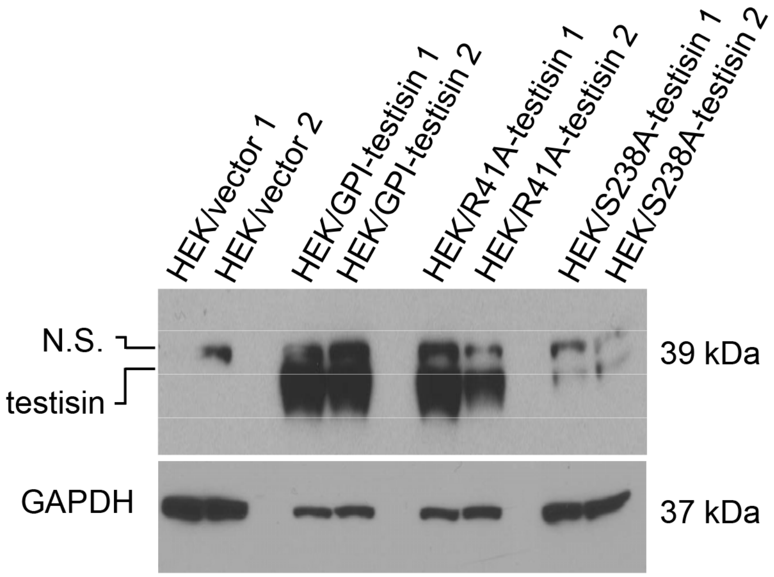

Supplementary Figure S2: Expression of full length human testisin and testisin mutants in HEK293T cells. Two independent pools of HEK293T cells each stably expressing full-length GPI-testisin (HEK/GPI-testisin), the R41A-testisin 'zymogen-locked' inactive mutant (HEK/R41A-testisin), the S238A-testisin inactive catalytic mutant (HEK/S238A-testisin), or transfected with vector alone (HEK/vector) were generated as described in the Materials and Methods. Immunoblot analysis using an anti-testisin antibody revealed expression of wild-type and mutant proteins at ~39 kDa. N.S. indicates a slower migrating non-specific band detected by this antibody. The S238A-testisin mutant was poorly detected compared to full-length GPI-testisin or the R41A-testisin mutant for unknown reasons, but possibly due to reduced stability of the expressed protein or reduced antibody affinity for this mutant. The blot was reprobed with anti-GAPDH antibody to assess protein loading and is representative of at least two independent experiments.

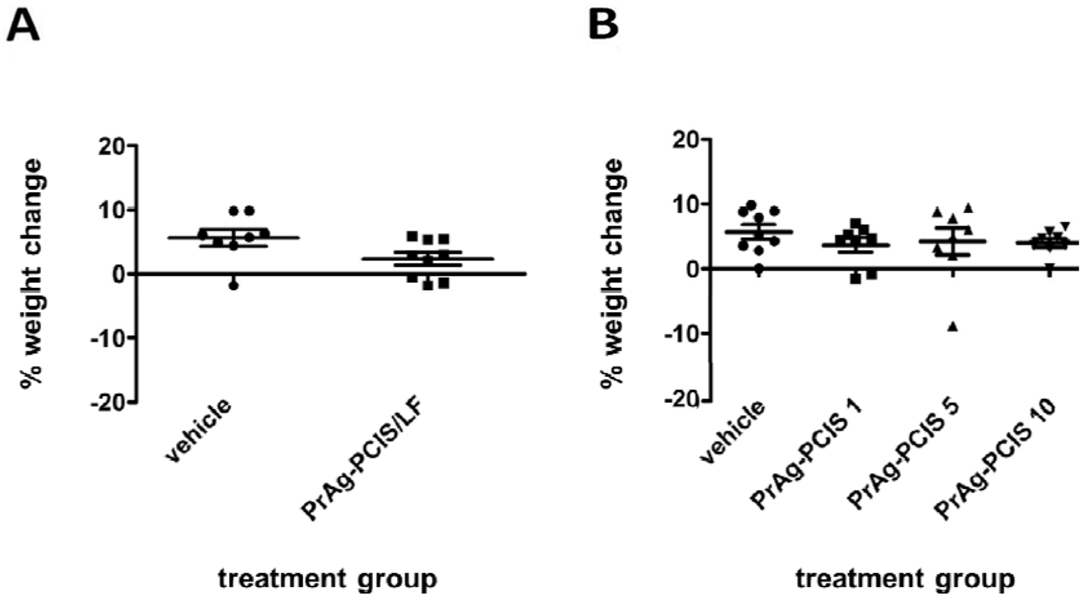

Supplementary Figure S3: No significant change was observed in whole body mouse weights due to treatment with PrAg-PCIS toxin. Mouse body weights were measured every 2–3 days throughout each animal experiment presented in Figure 7. **A.** The % change in weight of each mouse in Figure 7A from the day before treatment (day 10) to the last day of the experiment (day 24) $[(W_{day24}-W_{day10}/W_{day10})\times 100]$. **B.** The % change in weight of each mouse in Figure 7C from the day before treatment (day 12) to the last day of the experiment (day 28) $[(W_{day28}-W_{day12}/W_{day12})\times 100]$.
